# A Necessary Role for Cyclin D2 Induction During Colon Cancer Progression Mediated by L1

**DOI:** 10.3390/cells13211810

**Published:** 2024-11-02

**Authors:** Arka Saha, Nancy Gavert, Thomas Brabletz, Avri Ben-Ze’ev

**Affiliations:** 1 Department of Molecular Cell Biology, Weizmann Institute of Science, Rehovot 7610001, Israel; arka.saha@weizmann.ac.il (A.S.); nancy.gavert@weizmann.ac.il (N.G.); 2 Department of Experimental Medicine I, Nikolaus-Feibiger-Center for Molecular Medicine, University of Erlangen-Nuernberg, 91054 Erlangen, Germany; thomas.brabletz@fau.de

**Keywords:** colon cancer, L1, cyclin D2, invasion and liver metastasis

## Abstract

The cell adhesion molecule L1CAM (L1), mainly known for its function in brain cells, is a Wnt/β-catenin signaling target gene in colorectal cancer (CRC) cells, where it promotes invasion and liver metastasis. We interrogated which genes are expressed at increased levels in human CRC tissue and induced in CRC cell lines overexpressing L1. We found increased cyclin D2 levels in CRC tissue and LS 174T and HCT 116 human CRC cells overexpressing L1. Increased cyclin D2 in CRC cells was associated with higher proliferation rates, faster motility, tumorigenesis, and liver metastasis. The suppression of cyclin D2 expression by shRNA to cyclin D2 blocked the increase in these cellular properties of L1-expressing cells. The overexpression of cyclin D2 in the absence of L1 also conferred tumorigenic properties similar to L1 expression. The pathways involved in the elevation of cyclin D2 by L1 include NF-κB, Akt, and β-catenin signaling but not the Erk pathway. We found that in a significant percentage of human CRC tissue samples, cyclin D2 is expressed at high levels in the nuclei of cancer cells. At the same time, the adjacent normal mucosa was negative for cyclin D2 staining. The results suggest that the increased cyclin D2 expression by L1 is required to induce proliferative, motile tumor development in CRC tissue and can serve as a diagnostic marker and a target for CRC therapy.

## 1. Introduction

The overactivation of the Wnt/β-catenin signaling pathway and the transcription of downstream β-catenin-TCF target genes is a characteristic hallmark of colorectal cancer (CRC) progression [1,2,3,4]. We identified members of the L1CAM (L1) family of adhesion receptors (L1 and NrCAM) as target genes of the Wnt/β-catenin pathway whose expression is increased in CRC tissue [5,6]. L1 overexpression in human CRC cells confers increased proliferation, motility, tumorigenesis, and liver metastasis [6,7,8]. In CRC tissue, L1 is expressed exclusively at the invasive edge of the tumor but not in adenocarcinoma and the central, more differentiated tumor areas [6]. We searched for genes and signaling mechanisms required for L1-mediated CRC progression. We derived data sets of differentially expressed genes, both upregulated and downregulated, for L1-expressing LS 174T CRC cells and a set of CRC tissue samples [9]. We tested several such genes in previous studies and detected secreted growth factor receptors [10], ECM-modifying proteins [8,11,12,13,14], and stem cell markers [9] in CRC cells overexpressing L1 and demonstrated their requirement for enhanced CRC development [8,9,10,11,12,13,14,15,16]. In this study, we compared the upregulated genes in L1-overexpressing LS 174T cells to those that were increased in human CRC tissue to determine which genes are induced in both cases. We observed an increased expression of the cell cycle-regulating *cyclin D2* gene. 

Cyclin D2 belongs to the D-type cyclins that regulate G1/S transition in the cell cycle and define cell cycle progression and exit [17,18,19]. The aberrant expression and activity of cyclin D2 were suggested to lead to overgrowth and increased tumorigenesis [20,21,22,23,24,25]. Since previous studies have shown that *cyclin D2* is the most common cell cycle-regulating gene induced in CRC tissue [26,27] and correlates with increased liver metastasis, [28] we wished to determine whether L1 enhances tumorigenic properties in CRC cells by upregulating cyclin D2 expression.

## 2. Materials and Methods

### 2.1. Cell Culture and Reagents

The colorectal carcinoma (CRC) cell lines HCT 116 (ATCC CCL-247) and LS 174T (ATCC CL-188) were grown in RPMI-1640 (Gibco, Thermo Fisher Scientific, Paisley, UK) supplemented with 10% Fetal Bovine Serum (FBS) (Gibco, Thermo Fisher Scientific, Paisley, UK) and 1% penicillin/streptomycin solution (Gibco, Thermo Fisher Scientific, Paisley, UK). LS 174T and HCT 116 cells stably expressing human L1 (full length) were maintained in RPMI-1640 medium containing 10% FBS and 1% penicillin/streptomycin solution, supplemented with 800 µg/mL G418 solution (Sigma-Aldrich, St. Louis, MO, USA). LS 174T cells stably expressing L1 co-transfected with shp65 and IκB-SR cDNA were cultured in a selective medium supplemented with G418 (800 µg/mL) and puromycin dihydrochloride (10 µg/mL) (Sigma-Aldrich, St. Louis, MO, USA), as described previously [29]. Individual LS 174T cell clones stably expressing L1 and shcyclin D2 were maintained in a selective RPMI-1640 medium containing both G418 (800 µg/mL) and puromycin dihydrochloride (5 µg/mL) (Sigma-Aldrich, St. Louis, MO, USA). LS 174T cells stably expressing cyclin D2 were maintained in a selective RPMI-1640 medium supplemented with G418 (800 µg/mL). Individually isolated clones (LS 174T-L1 cl2; LS 174T-L1+ shcyclin D2 cl1 and cl2, and LS 174T-cyclin D2 cl1 and cl2) were used in this study. 

LS 174T-L1 and LS 174T pcDNA3 (empty vector) cells were treated with 1 µM of Selumetinib (MEK inhibitor) (Selleck Chemicals, Sylvanfield Drive, Houston, TX, USA) [13] for 24 h. LS 174T-L1 and LS 174T pcDNA3 (empty vector) cells were also treated with 5 µM of the Akt inhibitor MK2206 (Selleck Chemicals, Sylvanfield Drive, Houston, TX, USA) [13] for 24 h. In some experiments, LS 174T-L1 and LS 174T pcDNA3 (empty vector) cells were treated with 30 mM LiCl (Sigma-Aldrich, St. Louis, MO, USA), an inhibitor of glycogen synthase kinase 3 beta (GSK3β) for 24 h [13,30]. Cells treated with 30 mM NaCl (Sigma-Aldrich, St. Louis, MO, USA) served as the control [13]. In addition, in some experiments, LS 174T-L1 and LS 174T pcDNA3 (empty vector) cells were treated overnight with 10 µM of MG132 (Calbiochem, Burlington, MA, USA). 

### 2.2. Plasmids

The L1 [31], shp65, and IκB-SR cDNA used were described previously [29]. The Rc/CMV cyclin D2-HA plasmid was a gift from Philip Hinds (Addgene plasmid # 8950; http://n2t.net/addgene:8950; RRID:Addgene_8950, accessed on 19 July 2024) [32]. ShRNA to cyclin D2 sequences were ligated to pSUPER.puro plasmids per the manufacturer’s instructions (pSUPER. puro RNAi System, OligoEngine, Seattle, WA, USA). The shRNA to cyclin D2 target sequences are described in Table 1.

### 2.3. Transfection, Cell Motility, and Cell Proliferation Assays

LS 174T cells were transfected with pcDNA3-L1 as described [6,29,31]. ShRNA to cyclin D2 stably transfected into LS 174T-L1 cells, and LS 174T cells transfected with Rc/CMV cyclin D2-HA were performed using the Xfect™ reagent (TaKaRa Bio Inc., San Jose, CA, USA) as per the manufacturer’s instructions. The cell proliferation assay was performed by culturing 5000 cells in 12-well plates containing RPMI-1640 and 0.5% FBS. Cell counting was used to determine cell proliferation for six days. Graphs were plotted using the GraphPad Prism v9.2 software (San Diego, CA, USA, www.graphpad.com) to determine the cell numbers daily for six days.

Cell motility was determined by the “scratch wound” method, as described [14]. Briefly, 10^5^ cells were cultured in 12-well plates to form a confluent monolayer. Micropipette tips were used to create the “wounds” in the monolayer. In total, 2.5 μg/mL of Mitomycin C was added to inhibit cell proliferation. Images of the wounds were taken at 0 h and 24 h after wounding using a Nikon T2i inverted microscope (Nikon, Melville, NY, USA). The percentage of wound closure was calculated using the FIJI software (v.1.53c, NIH, Bethesda, MD, USA), and graphs were constructed using the GraphPad Prism v9.2 software (San Diego, CA, USA, www.graphpad.com).

### 2.4. Immunofluorescence

Immunofluorescence was performed on cells cultured on coverslips coated with poly-L-lysine (Sigma-Aldrich, St. Louis, MO, USA). Cells were permeabilized using 0.5% Triton X-100 and then fixed with 4% paraformaldehyde. Cells treated with 5% horse serum to block non-specific binding to cells were subsequently incubated with the rabbit polyclonal anti-L1 antibody (full length), recognizing the extracellular and intracellular domains of L1 (provided by Dr. V. Lemmon, University of Miami, Miami, FL, USA) [29,33] diluted 1:200 and mouse anti-cyclin D2 (DCS-3, Santa Cruz Biotechnology Inc., Dallas, TX, USA) diluted 1:200. The Alexa Flour-688-tagged goat anti-mouse IgG (ABCAM, Trumpington, Cambridge, UK) and Cy3-tagged goat anti-rabbit IgG (Jackson Immunoresearch Laboratories, West Grove, PA, USA) were used as secondary antibodies at a dilution of 1:2000. Nuclei were stained with 5 µg/mL 4′-6-diamidino-2-phenylindole (DAPI, Sigma-Aldrich, St. Louis, MO, USA). Immunofluorescence signals were detected by the Zeiss LSM 800 confocal microscope, and the images were captured using the ZEN imaging software (v2.3, blue edition) (Carl Zeiss Microscopy GmbH, Jena, Germany). 

### 2.5. Western Blotting

Whole-cell lysates were prepared from cells using RIPA cell lysis buffer supplemented with 1% protease inhibitors. The Pierce BCA protein assay (Thermo Fisher Scientific, Paisley, UK) was performed to determine the total protein concentration of the lysates. The 40 µg protein samples were resolved by 10% SDS-PAGE and transferred to 0.2 µm nitrocellulose membranes (Bio-Rad Laboratories, Haifa, Israel). Membranes were blocked with 5% skim milk (Sigma-Aldrich, St. Louis, MO, USA) in PBS supplemented with 0.5% Tween-20 (Bio-Basic Canada, Inc., Markham, ON, Canada). Membranes were subsequently probed with rabbit anti-L1 antibody against the full length L1 [29,33] diluted 1:2000; rabbit, polyclonal anti-cyclin D2 (10934-1-AP, Proteintech, Rosemont, IL, USA) diluted 1:250; rabbit anti-phospho−GSK3α/β (Ser21/9) (Cell Signaling Technologies, Beverly, MA, USA) diluted 1:1000; mouse anti-phospho ERK1/2 (Sigma-Aldrich, St. Louis, MO, USA), obtained from Prof. Yifat Merbl, Department of Immunology, Weizmann Institute of Science, Rehovot, Israel, diluted 1:1000; rabbit anti-phospho-Akt, s473, 4060, clone D9E (Cell Signaling Technologies, Beverly, MA, USA) diluted 1:1000; anti-rabbit NF-κB p65, sc-109 (Santa Cruz Biotechnology, Inc., Dallas, TX, USA) diluted 1:2000; anti-rabbit phospho-IκBα #2859 (Cell Signaling Technologies Inc., Danvers, MA, USA) diluted 1:1000; and anti-mouse anti-β-tubulin (Sigma-Aldrich), diluted 1:5000. Membranes were incubated at room temperature for 2 h with goat anti-mouse and anti-rabbit HRP-conjugated secondary antibodies (ABCAM, Trumpington, Cambridge, UK) diluted 1:2000. Signals were detected by the ECL chemiluminescence method (Amersham Biosciences, Buckinghamshire, UK), and bands were visualized by the ChemiDoc MP imaging system (Bio-Rad Laboratories, Haifa, Israel). Bands showing cyclin D2 expression were quantified using the ImageJ v1.54 software and normalized against the loading control by the Western blot quantification protocol [34]. Briefly, individual bands were quantified using the gel analysis tool in the ImageJ software (v.1.54, NIH, Bethesda, MD, USA). pcDNA3-transfected LS174T cells were used as the experimental control. The quantified blots were then normalized against tubulin, and the relative integrated densities were calculated and presented as a figure.

### 2.6. Quantitative RT-PCR

The Bio-Tri reagent (Bio-Lab, Jerusalem, Israel) was used to isolate total RNA from cells according to the manufacturer’s instructions. As instructed, cDNA was synthesized from the isolated RNA using the qScript cDNA Synthesis Kit (Quantabio, Beverly, MA, USA) [35]. Quantitative real-time PCR was performed with 200 ng/µL of cDNA using the Fast SYBR™ green master mix (Applied Biosystems™, Thermo Fisher Scientific Inc., Vilnius, Lithuania) as described [11]. Gene expression signals were recorded by the QuantStudio 3 design and analysis software v1.5.3 (Thermo Fisher Scientific, Waltham, MA, USA). Fold changes in gene expression were calculated by the ΔΔCT method, and graphs were plotted using the GraphPad Prism v9.2 software (GraphPad Software, Boston, MA, USA). The primers used for the quantitative RT-PCR analysis are shown in Table 2.

### 2.7. Tumor Growth and Metastasis Assays

Tumor growth was determined using 4-week-old male, athymic nude mice (Foxn-1nu), as described [12,13]. In brief, 3 × 10^6^ cells were suspended in 100 µL of PBS and injected subcutaneously on the flanks of mice at different sites [13]. Tumor growth in mice was monitored for 14 days after the injection until the tumors reached 1 cm in diameter. The tumors were then excised, photographed, and weighed. Graphs were plotted using the GraphPad Prism 9.2 software, showing the tumor weight in milligrams.

The metastatic potential of cells was determined as the ability of cells to migrate from the spleen to the liver in mice. In brief, 20 µL of PBS containing 3 × 10^6^ cells was injected into the distal tip of the spleen of 4-week-old, male, athymic nude mice as described [13]. The peritoneal injection of 10 mg/mL ketamine hydrochloride (Ketavet®, Zoetis, NJ, USA) and 10 mg/mL xylazine (Sedaxylan, Phibro Animal Health Corporation, NJ, USA) was used to anesthetize the mice before injecting cells into the spleen. The mice were sacrificed after six weeks, and tumor formation at the site of injection in the spleen and the presence of liver metastasis were observed and photographed.

### 2.8. Ethics Approval

The animal studies were reviewed, approved, and supervised by the Weizmann Institute Animal Care and Use (IACUC) ethics committee.

### 2.9. Immunohistochemistry

Colorectal cancer tissue samples were provided by the archives of the Department of Pathology, Erlangen, Germany, and 38 paraffin-embedded human colon adenocarcinoma tissue samples and the adjacent normal tissues were used for immunohistochemical analysis as described [8]. Patient identity was anonymized, and informed consent for using archived formaldehyde-fixed, paraffin-embedded material was not required (in 2003). The use of these samples for immunohistochemical analysis was approved by the local ethics committee (approval #374-14). Briefly, 4 μm thick tissue sections were de-paraffinized and dehydrated, and antigen retrieval was performed with a 10 mM citrate buffer at pH 6 (citric acid monohydrate, Sigma-Merck, Darmstadt, Germany, #C-7129 and 0.05% Tween-20) in a pressure cooker maintained at 121 °C for 22 min. Tissues were then probed with the rabbit anti-cyclin D2 primary antibody (ABIN 7153389, Antikoerper online) diluted 1:1500 in Dako diluent with background-reducing components (#S3022 Dako, Jena, Germany) and incubated overnight at 4 °C. Slides were then washed with TBS containing 0.05% Tween-20 and developed with the EnVision system (Dako, Jena, Germany) and the DAB kit (Cell Signaling DAB substrate kit #8059S) for the visualization of signals as instructed by the manufacturer [6].

### 2.10. DNA Microarrays

DNA microarray analysis was performed on RNA from LS 174T cells expressing L1 and pcDNA3 (empty vector)-transfected LS 174T cells using Affymetrix 1.0st GeneChips at the Weizmann Institute Microarray facility. The microarray data were analyzed as described previously [9], and a list of genes upregulated in L1-expressing cells compared to pcDNA3-transfected LS 174T cells was compiled [14]. Gene expression data from a large cohort of colorectal cancer and normal tissue, which included 52 normal tissue samples, 182 primary tumors, and 30 metastasis cases, was used to determine the differential expression of the previously selected genes [9,36,37]. The list of genes upregulated in LS 174T-L1 cells and colorectal cancer tissue samples with a fold change greater than 1.5 is shown in Table 3.

### 2.11. Statistical Analysis

Statistical analysis of the data was performed with the GraphPad Prism 9.2.0 software. Statistical significance was determined by Student’s non-paired *t-test* tool, and the results with *p* < 0.05 were considered statistically significant and are mentioned in the graphs with asterisks.

## 3. Results

### 3.1. Increased Expression of Cyclin D2 in Human CRC Tissue and L1-Overexpressing CRC Cell Lines

**Figure 1:**
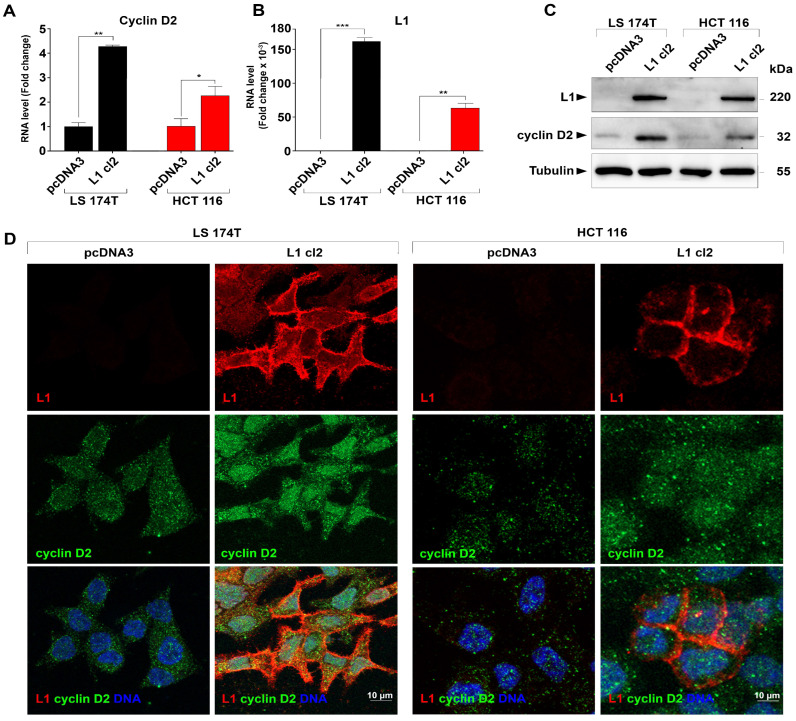
Induction of cyclin D2 expression in human CRC cell lines transfected with L1. (**A**) Increased *cyclin D2* RNA levels in LS 174T and HCT 116 human CRC cell lines overexpressing L1 as determined by RT-PCR. (**B**) The level of *L1* RNA in the CRC cell lines described in (**A**) was determined by RT-PCR. Cell lines were stably transfected with either L1 (L1 cl2) or the empty pcDNA3 plasmid (pcDNA3). (**C**) Western blots of the cell lines described in (**A**) show increased cyclin D2 protein levels in CRC cell lines overexpressing L1 (cyclin D2). (**D**) Immunofluorescent labeling of cyclin D2 (green) and L1 (red) in the CRC cell lines described in (**A**). Nuclei were stained with DAPI (blue). * p < 0.05, ** p < 0.01, *** p < 0.001.

By DNA microarray analysis, we found that 40.6% of genes that are significantly induced in L1-overexpressing LS 174T cells (compared to control pcDNA3-transfected cells) are also elevated in a set of human CRC tissue samples compared to the normal adjacent colon tissue (Table 3) [9,10]. Among these genes, we identified the cell cycle regulator *cyclin D2* (Table 3). RT-PCR and Western blot analysis of cyclin D2 identified an increased expression of *cyclin D2* RNA (Figure 1A) and proteins (Figure 1C) in both LS 174T and HCT 116 human CRC cell lines transfected with L1 (Figure 1B). Immunofluorescence analysis with an anti-cyclin D2 antibody revealed an increased speckle-like organization and the localization of cyclin D2 in both the cytoplasm and especially in the nuclei of L1-expressing CRC cells (Figure 1D, L1 cl2). We concluded from the different analyses of RNA and protein levels and localization that L1 expression increased the level of cyclin D2, especially in the nuclei of human CRC cell lines.

### 3.2. Isolation of CRC Cell Clones Overexpressing Cyclin D2 or L1 with Suppressed Levels of Cyclin D2

**Figure 2:**
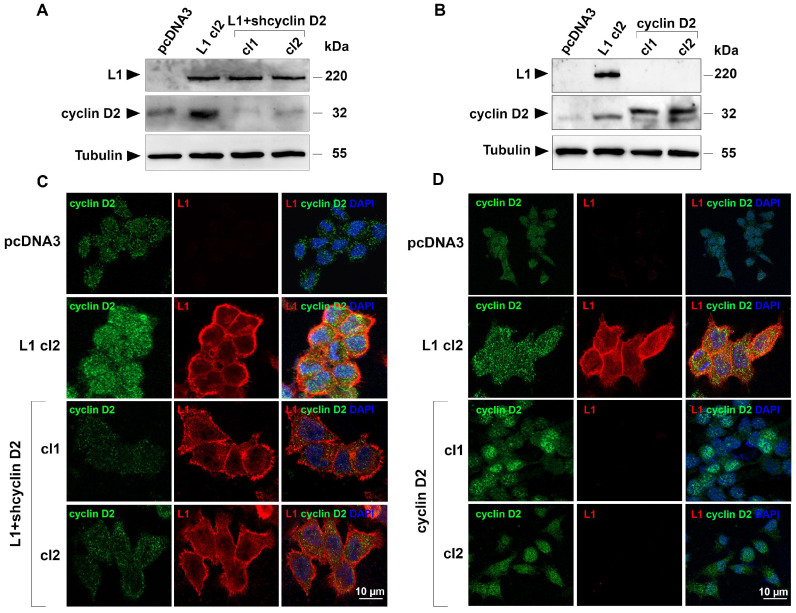
Isolation of CRC cell lines expressing varying levels of cyclin D2. (**A**) The levels of cyclin D2 were determined by Western blot in LS 174T CRC cells expressing L1 (L1 cl2) or the empty plasmid (pcDNA3). Cyclin D2 levels were suppressed using shRNA sequences compared to cyclin D2, and individual clones with reduced cyclin D2 levels were isolated (L1+shcyclin D2 cl1 and cl2). The levels of tubulin were used to determine the equal loading of the gels. (**B**) LS 174T cells were stably transfected with a cyclin D2-expressing plasmid, and clones overexpressing cyclin D2 (cyclin D2 cl1 and cl2) were isolated. (**C**) The localization and levels of cyclin D2 (green) and L1 (red) were determined in the LS 174T CRC cell clones described in (**A**) by immunofluorescence with antibodies compared to cyclin D2 and L1. (**D**) Immunofluorescent localization and the expression levels of cyclin D2 (green) and L1 (red) in the LS 174T CRC cell clones overexpressing cyclin D2 are shown in (**B**). Nuclei were stained with DAPI (blue).

To investigate the involvement of cyclin D2 in tumor development of L1-expressing human CRC cells, we isolated LS 174T cells, stably transfected with L1 in which the levels of endogenous cyclin D2 were suppressed using shRNA constructs compared to cyclin D2 (Figure 2A, L1+shcyclin D2, cl1, and cl2). Such cells displayed a reduced level of cyclin D2 by immunofluorescent staining with an anti-cyclin D2 antibody (Figure 2C). To investigate the effects of increased cyclin D2 expression on CRC development, independently of L1 expression, we also isolated LS 174T cell clones overexpressing cyclin D2 in the absence of L1 (Figure 2B, cyclin D2 cl1 and cl2). Cells overexpressing cyclin D2 showed an almost exclusive protein localization in the nuclei of cyclin D2-transfected cells (Figure 2D). With these CRC cell clones, we progressed to define their proliferation, motility, and tumorigenic and metastatic properties.

### 3.3. Increased Proliferation and Motility of CRC Cells Expressing High Levels of Cyclin D2 in the Presence or Absence of L1

**Figure 3:**
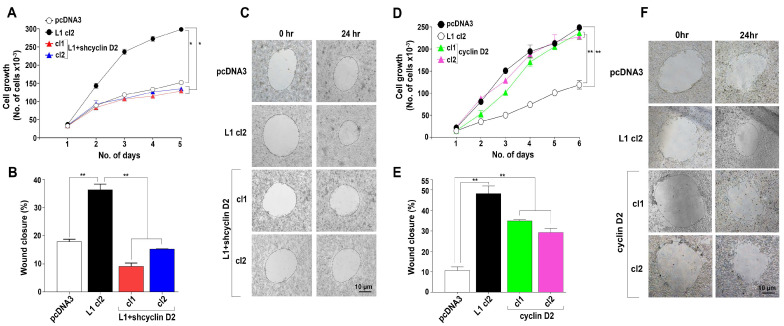
Proliferative and motile properties of CRC cell clones expressing L1 in which cyclin D2 levels were suppressed and in CRC cell clones overexpressing cyclin D2. (**A**) The proliferation of LS 174T cell clones overexpressing the empty plasmid (pcDNA3), L1 (L1 cl2), and L1 plus shRNA compared to cyclin D2 (L1+shcyclin D1 cl1, and cl2) was determined by seeding cells in serum-free medium. The number of cells was determined each day over five days by counting the cell number. (**B**) The motile properties of the different LS 174T cell clones described in (**A**) were determined by introducing a scratch wound in the confluent monolayers of cells, measuring the extent of wound closure after 24 h by photographing the same areas (**C**) and determining the denuded regions left. (**D**) The proliferation of LS 174T CRC cells overexpressing cyclin D2 (cyclin D2 cl1 and cl2) was determined in cells cultured in serum-free medium and by counting the cell number over six days as described in (**A**). (**E**,**F**) The motile properties of LS 174T cell clones overexpressing cyclin D2 (cyclin D2 cl1 and cl2) were determined as described in (**B**,**C**) for cell clones displaying suppressed cyclin D2 levels. * p < 0.05, ** p < 0.01.

To detect the changes in the rate of cell proliferation of CRC cell clones with varying levels of cyclin D2, we cultured the LS 174T cell clones described in Figure 2 for six days under stressful conditions (medium without serum) and determined the cell number each day [10]. The results presented in Figure 3A show that L1-expressing CRC cell clones in which the endogenous levels of cyclin D2 were suppressed by shRNA compared to cyclin D2 displayed a decreased ability to proliferate, similar to control pcDNA3-transfected cells (Figure 3A, L1+shcyclin D2 cl1, and cl2 compared to pcDNA3 and L1 cl2). The powerful influence of cyclin D2 on CRC cell proliferation was observed in LS 174T CRC cell clones transfected with cyclin D2 in the absence of L1 that displayed an increase in proliferation over six days, similar to that induced by L1 (Figure 3D, cyclin D2 cl1, and cl2, compared to L1 cl2).

Next, we determined the motile properties of these CRC cell clones by measuring their ability to close an artificial wound introduced in a confluent monolayer 24 h after wounding [10] (Figure 3B,C,E,F). As shown in Figure 3B,C, the motility of L1+shcyclin D2 clones was dramatically reduced when compared to L1-expressing cells. CRC cell clones overexpressing cyclin D2 in the absence of L1 displayed an increase in their wound closure ability, similar to cells overexpressing L1 (Figure 3E,F). Taken together, we conclude that a high expression of cyclin D2 in CRC cells overexpressing L1 is required for increased proliferation and cell motility.

### 3.4. Increased Tumorigenesis and Liver Metastasis of CRC Cells Mediated by L1 Requires a High Level of Cyclin D2 Expression 

**Figure 4:**
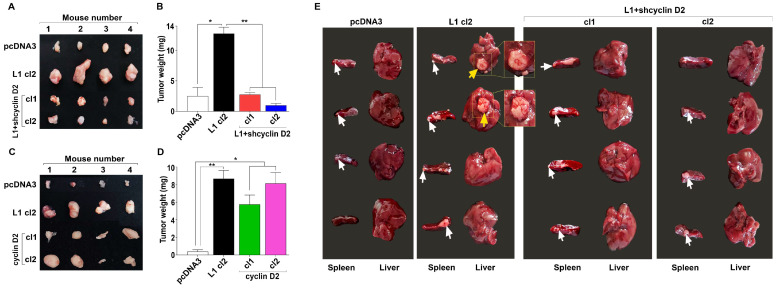
The tumorigenic and liver metastatic capacity of CRC cells expressing varying cyclin D2 levels. (**A**) A group of 4 male nude mice was injected (each mouse) in the flanks with LS 174T cells expressing pcDNA3, L1 (L1 cl2), and L1+shcyclin D2 (cl1 and 2). After three weeks, the tumors were excised and photographed. (**B**) Tumor weight was determined for each cell clone described in (**A**). (**C**) Four nude mice were injected (each mouse) in the flanks with LS 174T expressing pcDNA3, L1 (L1 cl2), and cyclin D2 (cyclin D2 cl1, and cl2). The tumors formed after three weeks were excised and photographed, and their weight was determined (**D**). (**E**) Groups of 4 male nude mice (4 weeks old) were injected in the tip of their spleen with cell clones described in (**A**). After six weeks, the spleens and the livers were excised and photographed. The white arrows mark tumor growth at injection sites in the spleens. The yellow arrows mark liver metastases. * p < 0.05, ** p < 0.01.

We wished to determine the tumorigenic and liver metastatic capacities of the LS 174T CRC cell clones described in Figure 2A,B. Cells were injected into the flanks of nude mice, and tumor growth was determined two weeks after injection. The size and weight of the tumors significantly increased in CRC cell lines overexpressing L1 (Figure 4A,B) compared to pcDNA3-transfected cells. This increase in tumor size and weight required the induction of cyclin D2 by L1 since the suppression of cyclin D2 levels by shRNA compared to cyclin D2 blocked the increased tumor growth induced by L1 (Figure 4B, L1+shcyclin D2 cl1, and cl2, compared to L1 cl2). Cyclin D2 overexpression without L1 in LS 174T CRC cells could also enhance tumor growth (Figure 4C,D), suggesting that increased cyclin D2 expression is a powerful pro-tumorigenic event that can confer tumor growth in human CRC cells.

We wished to determine the metastatic abilities of the LS 174T human CRC cell clones expressing L1 in which the levels of endogenous cyclin D1 were suppressed. Cells of the various clones (Figure 2A) were injected into the tip of the spleen in male nude mice, and the formation of liver metastases was assessed six weeks later. The results shown in Figure 4E show that an increase in cyclin D2 is required to confer liver metastasis in L1-expressing cells, and the suppression of cyclin D2 levels blocked the metastatic capacity of L1-transfected cells.

### 3.5. Signaling Pathways Involved in the Induction of Cyclin D2 in CRC Cell Lines by L1

**Figure 5:**
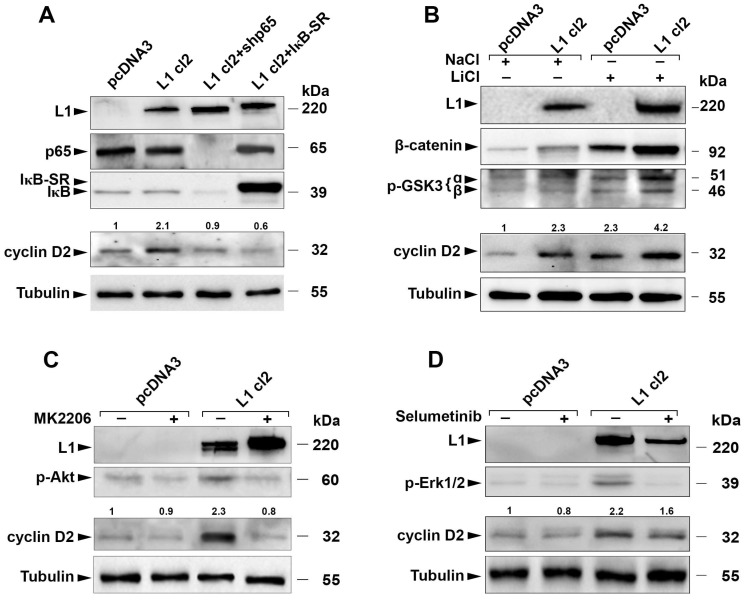
The L1-mediated elevation in cyclin D2 involves the NF-κB, Wnt/β-catenin, and Akt signaling pathways. (**A**) The NF-κB pathway was inhibited in L1-expressing LS 174T cells by suppressing the levels of the p65 subunit of NF-κB (L1 cl2+shp65) or by expressing the IκB super repressor (L1 cl2+IκB-SR). The levels of cyclin D2 in these cell clones and control cells transfected with empty plasmid (pcDNA3) and cells transfected with L1 (L1 cl2) were determined by Western blot with the corresponding antibodies. The numbers above the cyclin D2 bands represent densitometric values normalized to pcDNA3-transfected cells after normalizing to the loading control tubulin band. (**B**) The Wnt/β-catenin pathway was activated by inhibiting the degradation of β-catenin controlled by GSK3β using 30 mM LiCl for 24 h to treat the cells. NaCl treatment served as a control for LiCl treatment. The numbers above the cyclin D2 bands represent the densitometric values normalized to pcDNA3-transfected cells after normalizing to the loading control tubulin band. (**C**) The Akt pathway was inhibited by treating the cells overnight with the Akt inhibitor MK2206, and the levels of cyclin D2 were determined as described in (**A**,**B**). (**D**) The signaling by Erk was inhibited by the overnight treatment of pcDNA3 and L1-expressing LS 174T cells using the Erk1/2 inhibitor Selumetinib. The levels of cyclin D2 were determined by Western blot as described in (**C**).

In previous studies, we investigated the signaling pathways by which L1 regulates the expression of downstream target genes [9,10,11,12,13,14,15,16,30,38,39]. Here, we determined which pathways are involved in the induction of cyclin D2 in human CRC cells by L1. The inhibition of NF-κB signaling by an shRNA to the p65 subunit of NF-κB or by the overexpression of a super-repressor of IκB (IκB-SR) reduced the level of cyclin D2 in LS 174T CRC cells expressing L1 (Figure 5A, cyclin D2, Supplementary Figure S1A), suggesting that this pathway is involved in the induction of cyclin D2 by L1. To investigate the possible involvement of the Wnt/β-catenin pathway in the increase in cyclin D2 by L1, we blocked the turnover of β-catenin that involves GSK3β using the GSK3β inhibitor LiCl that induces increased GSK3β phosphorylation/inactivation (Figure 5B, p-GSK3α and β). The increase in the β-catenin level in LiCl-treated cells (Figure 5B, β-catenin) resulted in the elevation of cyclin D2 in L1-expressing cells (Figure 5B, cyclin D2, Supplementary Figure S1B), supporting the role of the Wnt/β-catenin pathway in the L1-mediated rise in cyclin D2. In addition, we found that by inhibiting the proteasomal degradation of β-catenin with the proteasomal inhibitor MG132, the cyclin D2 protein level was elevated in LS 174T CRC cells expressing L1, further supporting the involvement of the Wnt/β-catenin pathway in this process (Supplementary Figure S2A–C). We also determined the possible involvement of the Akt and Erk pathways in the cyclin D2 induction by L1. Using the Akt inhibitor MK2206 (Figure 5C, p-Akt) that reduces the level of p-Akt, we found that the increase in cyclin D2 by L1 was blocked (Figure 5C, cyclin D2, Supplementary Figure S1C). In contrast, using the Erk1/2 inhibitor Selumetinib that reduces the level of activated p-Erk1/2 (Figure 5D, p-Erk1/2), we found no significant effect on the induction of cyclin D2 by L1 in LS 174T CRC cells (Figure 5D, cyclin D2, Supplementary Figure S1D). These results suggest that the NF-κB, β-catenin, and Akt pathways are involved in the induction of cyclin D2 by L1 in CRC cells, while the Erk pathway has no significant impact on this process. 

### 3.6. Cyclin D2 Is Localized in the Nuclei of CRC Tissue but Not in the Adjacent Normal Tissue

**Figure 6:**
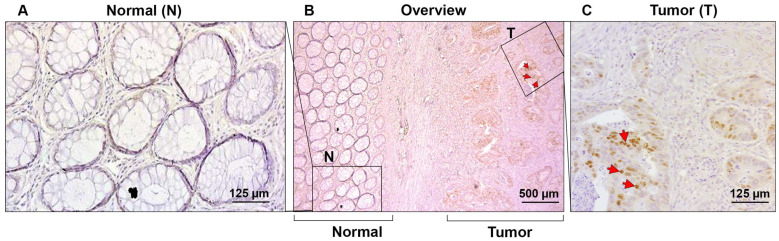
The localization of cyclin D2 in human CRC tissue. Paraffin-embedded formaldehyde-fixed tissue samples from 38 CRC patients were processed by immunohistochemistry to detect cyclin D2 localization. (**A**) The normal (N) mucosa was negative for cyclin D2 staining. (**B**) An overview of the area containing both normal (N) and tumor tissue (T). Cyclin D2 staining was observed in 34% of the CRC tissue samples. (**C**) Enlarged area of a tumor tissue (T) displaying nuclear staining for cyclin D2 (red arrows).

We wished to determine the localization and expression level of cyclin D2 in human CRC tissue. Immunohistochemical analysis with an antibody against cyclin D2 was conducted on 38 human formaldehyde-fixed CRC tissue samples. Intense nuclear staining was detected in cancer cells in 34% of the CRC tissue samples (Figure 6B,C, (T), red arrowheads). The adjacent normal mucosa was negative for cyclin D2 staining (Figure 6A,B (N)), with sometimes a very weak signal in individual cells. These results demonstrate that cyclin D2 is induced in CRC tissue and localized in cancer cell nuclei. 

## 4. Discussion

In this study, we found that the cell cycle regulator cyclin D2, which plays a crucial role in the transition from G1 to the proliferative stage of cells (S phase) [18,19], is induced in both human CRC tissue and in L1-expressing human CRC cell lines (LS 174T and HCT 116). This increase in cyclin D2 in LS 174T CRC cells is required to confer the tumorigenic and metastatic properties during L1-mediated tumor development. Since we have previously shown that HCT 116 cells are poorly tumorigenic and do not form liver metastases in mice [8], we could not conduct such experiments with HCT 116-L1 cells to further support the conclusions on phenotypes observed with LS 174T cells. Suppressing the level of cyclin D2 in L1-expressing cells blocks the pro-tumorigenic properties conferred by L1 in CRC cells. Conversely, the overexpression of cyclin D2 in CRC cells lacking L1 induces tumorigenic properties similar to L1, indicating that the rise in cyclin D2 levels is both necessary and sufficient for CRC progression. Cyclin D2 expression was observed by immunohistochemistry in 34% of the CRC tissue samples and was localized mainly in the nuclei of CRC tissue, and LS 174T cell clones transfected with cyclin D2, which is consistent with its role in the nucleus during the cell cycle regulation. Previous studies have suggested that cyclin D2 expression is related to poor prognosis in CRC patients [27], and an increase in cyclin D2 levels resulting in overproliferation was reported in various premalignant [40,41,42] and cancer tissues [24], including germ cell tumors [43], hematopoietic cells [21,25,44,45], granulosa cells [22,46], gastric tumors [47,48], and CRC [49]. An elevated cyclin D2 level was detected at the invasive edge of CRC tissue and was suggested as an independent predictor of hepatic metastasis [28,50]. We have previously shown that L1 is expressed only in cells at the leading edge of invasive CRC tissue [6], and we did not observe significant L1 and cyclin D2 co-localization in CRC tissues. Further experiments are warranted to investigate the lack of co-localization between cyclin D2 and L1 in CRC tissues. Studies with the *Apc^Min/+^* transgenic mouse model for CRC development showed that cyclin D2 is upregulated immediately after Apc loss in cells with deregulated Wnt/β-catenin signaling and that cyclin D2 deficiency reduces the proliferation and tumor burden after Apc loss [49,51]. In addition, the overexpression of cyclin D2 was reported to be the most common aberration in human colonic crypts [26]. Our studies show that increased Wnt/β-catenin signaling is involved in cyclin D2 induction by L1 overexpression, which agrees with these findings. In addition to the Wnt/β-catenin pathway, we have demonstrated that NF-κB signaling is essential for cyclin D2 induction by L1. We have previously unraveled the mechanisms of NF-κB activation by L1 [10,29] and have shown that blocking this signaling pathway inhibits the induction of cyclin D2 by L1. These studies showing the involvement of NF-κB signaling in L1-mediated cyclin D2 induction are supported by reports demonstrating that the activation of the *cyclin D2* gene proceeds through NF-κB in T cells [52].

The involvement of Akt signaling in the induction of cyclin D2 by L1 and the blocking of cyclin D2 expression by an inhibitor of Akt activation (MK2206) are supported by reports showing that the expression of cyclin D2 is down-regulated in B-cells by inhibiting the Akt pathway with BEZ-235 [25,53]. 

L1 is a 200–220 kDa transmembrane glycoprotein reported to undergo proteolytic cleavage that releases a soluble cytosolic intracellular cleavage fragment that can translocate to the nucleus and regulate gene expression [54,55,56,57]. Future studies will have to determine if this processing of L1 is involved in cyclin D2 induction, and which molecular interactions enable such signaling. This study shows that the induction of cyclin D2 expression by L1 in CRC cells is a critical step in tumor development and that the inhibition of the various signaling pathways involved in this process could provide therapeutic targets against CRC development.

## Figures and Tables

**Table 1:** ShRNA target sequences against cyclin D2 RNA.

Name	Sequence
shcyclin D2_1	GATCCCCAATTACCTGGACCGTTTCTTTCAAGAGAAGAAACGGTCCAGGTAATTTTTTTA
shcyclin D2_2	GATCCCCTGCTCCTCAATAGCCTGCATTCAAGAGATGCAGGCTATTGAGGAGCATTTTTA
shcyclin D2_3	GATCCCCTGAATTACCTGGACCGTTTTTCAAGAGAAAACGGTCCAGGTAATTCATTTTTA
shcyclin D2_4	GATCCCCTGACGGATCCAAGTCGGAGTTCAAGAGACTCCGACTTGGATCCGTCATTTTTA

**Table 2:** Primers used for qRT-PCR.

Gene Name	Forward	Reverse
*cyclin D2*	TCCTGGCCTCCAAACTCAAA	AAGTCATGAGGAGTGACAGC
*L1*	TCGCCCTATGTCCACTACACCT	ATCCACAGGGTTCTTCTCTGGG
*GAPDH*	GTCTCCTCTGACTTCAACAGCG	ACCACCCTGTTGCTGTAGCCAA

**Table 3:** List of genes overexpressed in tumor versus (vs) normal tissue in CRC cases and L1-overexpressing LS 174T versus (vs) pcDNA3-transfected cells.

Gene Symbol	Fold Change (LS 174T-L1 vs. pcDNA3)	*p*-Value	Fold Change (Tumor vs. Normal)	*p*-Value	Description
ARSE	3.08519	1.73 × 10^−19^	3.571821	5.5 × 10^−6^	arylsulfatase (chondrodysplasia punctata 1)
CEACAM5	2.45604	1.84 × 10^−12^	3.424527	0.019633	carcinoembryonic antigen-related cell adhesion molecule 5
C12orf5	2.07612	2.30 × 10^−9^	3.348652	7.12 × 10^−7^	chromosome 12 open reading frame 5
ALDH3A1	1.9768	6.92 × 10^−9^	3.055874	0.000381	aldehyde dehydrogenase 3 family, member A1
CCND2	1.97026	1.24 × 10^−11^	3.009642	0.00179	cyclin D2
CDC25A	1.94782	1.18 × 10^−8^	2.978324	2.08 × 10^−14^	cell division cycle 25 homolog A (S. pombe)
HSPE1	1.92853	5.95 × 10^−8^	2.657038	1.08 × 10^−16^	heat shock 10 kDa protein 1 (chaperonin 10)
ACTL8	1.92409	7.29 × 10^−8^	2.633271	0.002426	actin-like 8
INPP5D	1.85847	1.02 × 10^−6^	2.60498	1.01 × 10^−11^	inositol polyphosphate-5-phosphatase, 145 kDa
CEACAM6	1.79795	1.12 × 10^−5^	2.586789	1.64 × 10^−11^	carcinoembryonic antigen-related cell adhesion molecule 6 (non-specific cross reacting antigen)
POLR3G	1.79191	6.39 × 10^−7^	2.553357	3.87 × 10^−17^	polymerase (RNA) III (DNA-directed) polypeptide G (32 kD)
CACYBP	1.77876	1.55 × 10^−8^	2.502908	6.64 × 10^−12^	calcyclin-binding protein
PLK2	1.75368	3.33 × 10^−6^	2.490094	0.000479	polo-like kinase 2 (Drosophila)
RRM2	1.72587	2.49 × 10^−6^	2.402542	3.72 × 10^−16^	ribonucleotide reductase M2 polypeptide
CD70	1.70231	6.98 × 10^−6^	2.399618	0.000594	CD70 molecule
TNFSF9	1.70175	6.14 × 10^−6^	2.361174	2.98 × 10^−5^	tumor necrosis factor (ligand) superfamily, member 9
CHORDC1	1.67797	9.30 × 10^−6^	2.279949	3.45 × 10^−6^	cysteine and histidine-rich domain (CHORD)-containing 1
PLLP	1.66414	1.47 × 10^−5^	2.258648	0.004717	plasma membrane proteolipid (plasmolipin)
ABCE1	1.65432	1.80 × 10^−5^	2.087956	6.11 × 10^−18^	ATP-binding cassette, sub-family E (OABP), member 1
CDC6	1.64504	1.56 × 10^−5^	2.017744	2.6 × 10^−17^	cell division cycle 6 homolog (S. cerevisiae)
RRS1	1.63547	2.49 × 10^−5^	2.007606	4.59 × 10^−18^	RRS1 ribosome biogenesis regulator homolog (S. cerevisiae)
GDF15	1.60155	3.27 × 10^−5^	1.912974	7.1 × 10^−24^	growth differentiation factor 15
KCNN4	1.60087	8.16 × 10^−5^	1.908127	1.31 × 10^−16^	Potassium intermediate/small conductance calcium-activated channel, subfamily N, member 4

## Data Availability

The data presented in this study are available on request from the corresponding author.

## Supplementary Materials

The following supporting information can be downloaded at https://www.mdpi.com/article/10.3390/cells13211810/s1. Figure S1: Cyclin D2 protein expression represented as average integrated density in different molecular pathways involved in L1-mediated CRC development; Figure S2: Cyclin D2 protein levels in LS 174T pcDNA3 and L1 cl2-expressing cells after treatment with the proteasome inhibitor MG132.
