# Supplementary material for: A Necessary Role for Cyclin D2 Induction During Colon Cancer Progression Mediated by L1
Source: Cells. 2024 Nov 2;13(21):1810. doi: 10.3390/cells13211810 (PMC11544798; doi:10.3390/cells13211810)
Supplement: Supplementary file 1 [file cells-13-01810-s001.zip › cells-3199809-supplementary.pdf]

Supplementary Material

A Necessary Role for Cyclin D2 Induction During Colon Cancer Progression mediated by L1

Arka Saha [1], Nancy Gavert [1], Thomas Brabletz [2] and Avri Ben-Ze'ev [1*]

[1] Department of Molecular Cell Biology, Weizmann Institute of Science, Rehovot 7610001, Israel; arka.saha@weizmann.ac.il (A.S.); nancy.gavert@weizmann.ac.il (N.G.)

[2] Department of Experimental Medicine I, Nikolaus-Feibiger-Center for Molecular Medicine, University of Erlangen-Nuernberg, 91054 Erlangen, Germany; thomas.brabletz@fau.de

* Correspondence: avri.ben-zeev@weizmann.ac.il;

Figure S1: Cyclin D2 protein levels related to different molecular pathways involved in L1-mediated CRC development presented as average integrated densities from SDS-PAGE gels.

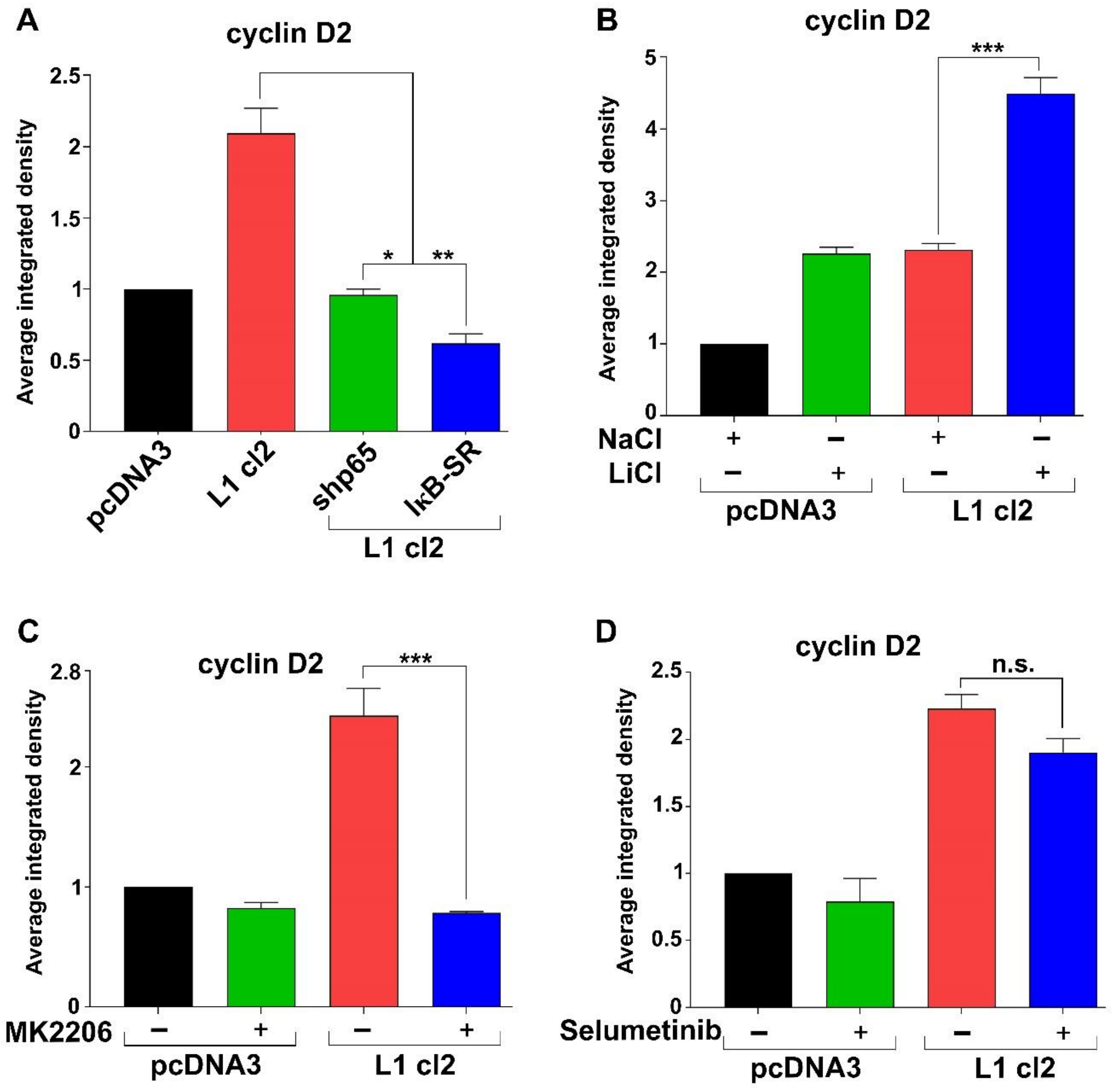

A: The average integrated density of cyclin D2 expression calculated from the western blots shown in Figure 5A. Reduced expression of cyclin D2 when the NF-κB pathway was inhibited in L1-expressing LS 174T cells by suppressing the levels of the p65 subunit of NF-κB (L1 cl2+shp65), or by expressing the IκB super repressor (L1 cl2+IκB-SR). **B:** The average integrated density of cyclin D2 expression calculated from the western blots of Figure 5B shows increased cyclin D2 levels by inhibiting the degradation of β-catenin by GSK3β using 30 mM LiCl for 24 hours in LS 174T-L1 cells. **C:** Densitometric analysis of cyclin D2 protein expression calculated from the western blot of Figure 5C shows reduced levels when the Akt pathway was inhibited with MK2206 in LS 174T-L1 cells. **D:** Densitometric

analysis of the western blot data in Figure 5D shows no significant change in cyclin D2 expression upon inhibition of the ERK pathway using Selumetinib in LS 174T-L1 cells. * *p* < *0.05, ** p < 0.01, ***p<0.001.*

Figure S2: cyclin D2 protein levels in LS 174T pcDNA3 and L1 cl2-expressing cells after treatment with the proteasome inhibitor MG132.

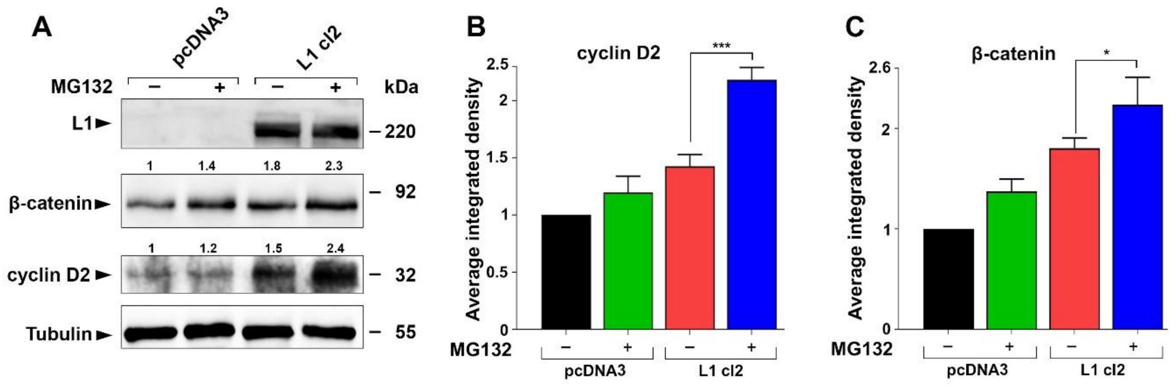

A: The Wnt/β-catenin pathway was enhanced by preventing the proteasomal degradation of β-catenin with MG132. This increased the cyclin D2 protein levels in LS 174T-L1 cells after treatment with MG132. The numbers above the cyclin D2 and β-catenin bands represent densitometric values normalized to pcDNA3-transfected cells after normalizing to the loading control tubulin. **B and C**: The densitometric values of cyclin D2 and β-catenin were calculated from the blots in (A), representing average integrated densities from three experiments. * *p < 0.05, *** p<0.001.*
